# The double burden of diabetes and global infection in low and middle-income countries

**DOI:** 10.1093/trstmh/try124

**Published:** 2018-12-04

**Authors:** Susanna Dunachie, Parinya Chamnan

**Affiliations:** 1 Mahidol-Oxford Tropical Medicine Research Unit, Mahidol University, 3rd Floor, 60th Anniversary Chalermprakiat Building, 420/6 Ratchawithi Rd., Ratchathewi District, Bangkok, Thailand; 2 Centre for Tropical Medicine and Global Health, University of Oxford, Nuffield Department of Medicine Research Building, University of Oxford, Old Road campus, Roosevelt Drie, Headington, Oxford, United Kingdom; 3 The Peter Medawar Building for Pathogen Research, University of Oxford, South Parks Road, Oxford, United Kingdom; 4 Cardiometabolic Research Group, Department of Social Medicine, Sunpasitthiprasong Hospital, Tambon Nai Mueang, Amphoe Mueang Ubon Ratchathani, Chang Wat Ubon Ratchathani, Thailand

**Keywords:** dengue, diabetes, global infection, melioidosis, tropical, tuberculosis

## Abstract

Four out of five people in the world with diabetes now live in low- and middle-income countries (LMIC), and the incidence of diabetes is accelerating in poorer communities. Diabetes increases susceptibility to infection and worsens outcomes for some of the world’s major infectious diseases such as tuberculosis, melioidosis and dengue, but the relationship between diabetes and many neglected tropical diseases is yet to be accurately characterised. There is some evidence that chronic viral infections such as hepatitis B and HIV may predispose to the development of type 2 diabetes by chronic inflammatory and immunometabolic mechanisms. Helminth infections such as schistosomiasis may be protective against the development of diabetes, and this finding opens up new territory for discovery of novel therapeutics for the prevention and treatment of diabetes. A greater understanding of the impact of diabetes on risks and outcomes for infections causing significant diseases in LMIC is essential in order to develop vaccines and therapies for the growing number of people with diabetes at risk of infection, and to prioritise research agendas, public health interventions and policy. This review seeks to give an overview of the current international diabetes burden, the evidence for interactions between diabetes and infection, immune mechanisms for the interaction, and potential interventions to tackle the dual burden of diabetes and infection.

## Introduction

There are now 336 million people with diabetes living in low- and middle-income countries (LMIC).^1,2^ Diabetes increases susceptibility to infection and worsens outcomes for diseases such as tuberculosis (TB),^3^ and the under-recognised tropical disease melioidosis.^4,5^ Current international treatment guidelines for diabetes are based on research conducted in high-income countries focussed on preventing adverse cardiovascular outcomes and early death. There is a lack of evidence upon which to base guidelines for people living in LMIC, where there is an increased burden of infectious diseases compared with high income countries.

## Epidemiology of diabetes

**Figure 1.**
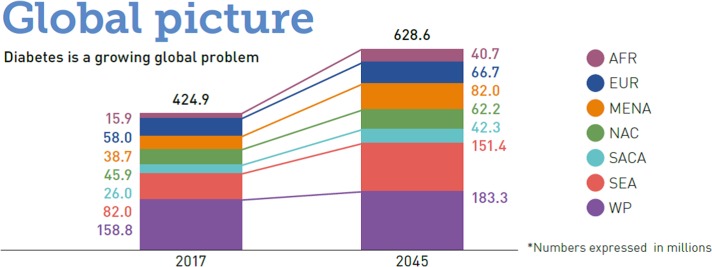
Predicted increased prevalence of diabetes from 2017 to 2045 by geographic region; AFR, sub-Saharan Africa; EUR, Europe; MENA, Middle East and North Africa; NAC, North America and the Caribbean; SACA, South and Central America; SEA, South East Asia including India; WP, Western Pacific including China, Indonesia and Australia. Reproduced with kind permission from the International Diabetes Federation World IDF Diabetes Atlas 2017^1^.

Diabetes has traditionally been viewed as a ‘disease of the wealthy’, mostly found among elderly people in developed countries. Now, however, diabetes affects every strata of society, and has become a fast-growing problem in poorer communities. The Global Burden of Disease (GBD) study estimated there were 1.4 million deaths worldwide from diabetes in 2016,^6^ representing a 31% increase from 2006. Of the estimated 425 million people with diabetes worldwide, four-fifths currently live in LMIC and increasing numbers of children and young adults have been diagnosed with the disease.^1^ This number is projected to increase to 629 million by 2045, and most of the rising burden will occur in LMIC (Figure 1). In addition, diabetes is more likely to be undiagnosed or poorly treated in LMIC.^1^

Data from the NCD Risk Factor Collaboration^2^ show that age-standardised diabetes prevalence in adults has increased or remained unchanged since 1980 in every country.^2^ Importantly, the burden of diabetes has increased faster in LMIC than in high-income countries.^2^ This rising prevalence of diabetes in LMIC is believed to be associated with many factors, including ageing populations, urbanisation, cultural and social changes, dietary changes, physical inactivity, changes in diagnostic criteria and screening practices, better treatment and survival, and increasing trends in overweight and obesity.

### Classification of diabetes

Table 1 shows the current classification of diabetes by WHO and the American Diabetes Association (ADA),^7,8^ which includes four clinical and aetiological classes: type 1 diabetes (T1DM), type 2 diabetes (T2DM), gestational diabetes mellitus, and other specific types of diabetes due to other causes. T1DM is caused by an autoimmune reaction which destroys the insulin-producing beta cells in the islets of the pancreas, leading to no or low production of insulin. T2DM is the most common type of diabetes, accounting for approximately 90% of all cases of diabetes worldwide.^1^ It is characterised by an inadequate production of insulin and an inability of the body to respond fully to insulin, defined as insulin resistance. It is important to note that assigning a type of diabetes to an individual is often reliant on the circumstances present and additional testing at the time of diagnosis, and that many patients with diabetes do not easily fit into a single class. In LMIC, it is often unknown which type of diabetes a person has, and therefore this review will use the general term ‘diabetes’, but >90% of patients with diabetes in LMIC are believed to have T2DM.^1^

**Table 1.** Aetiological classification of diabetes mellitus, adapted from WHO^7^ and the American Diabetes Association^8^

Type	Pathophysiology
Type 1 ▪ Autoimmune ▪ Idiopathic	Results from β-cell destruction, usually leading to absolute insulin deficiency
Type 2 ▪ Predominantly insulin resistance ▪ Predominantly insulin secretory defects	May range from predominantly insulin resistance with relative insulin deficiency to a predominantly secretory defect with or without insulin resistance
Other specific types	Results from other causes including genetic defects in β-cell function; genetic defects in insulin action; diseases of the exocrine pancreas; endocrinopathies; drug or chemical induced; infections
Gestational diabetes	Diagnosed during pregnancy (encompasses gestational impaired glucose tolerance and gestational diabetes mellitus)

### Risk factors of diabetes

Table 2 summarises the modifiable and non-modifiable risk factors for T2DM, which is a disease caused by a complex interplay between genetic and environmental factors.^9^ The rapid increase in the prevalence of diabetes over recent decades suggests that environmental and lifestyle factors might play an increasingly important role in the development of the disease.

**Table 2.** Modifiable and non-modifiable risk factors for type 2 diabetes (adapted from the International Diabetes Federation: a consensus on type 2 diabetes prevention)^9^

Modifiable risk factors	Non-modifiable risk factors
Overweight and obesity (central and total)	Age
Sedentary lifestyle	Sex
Adverse diet/dietary factors	Ethnicity
Smoking	Family history of type 2 diabetes
Intrauterine environment	History of gestational diabetes
Hypertension/use of antihypertensive medication	
Serum cholesterol	
Triglycerides	
Previously identified glucose intolerance	

## Diabetes and specific infections

It is generally recognised that people with diabetes are at increased risk of infection and worse outcomes,^10,11^ including diabetic foot infection, urinary tract infections (especially from *Escherichia coli)*, *Streptococcal* pneumonia, cellulitis (of which *Streptococcal* species are a common cause), *Candida* and *Mucor* invasive fungal infections, skin and surgical site infections. This review will focus on the evidence for increased risk to pathogens of particular significance in LMIC, with the findings summarised in Box 1

**Box 1.** Relationship between diabetes and susceptibility/increased disease severity for significant pathogens in low- and middle-income countries. See text for discussion of evidence.

Established increased susceptibility/severity (several good quality studies)	Probable increased susceptibility/severity (some evidence)	Further evidence of interaction needed (single or conflicting reports, or lack of data)	Potential inverse interaction (single studies or more)
*Mycobacterium tuberculosis* (TB)	*Mycobacterium leprae* (leprosy)	*Plasmodium falciparum* (malaria)	*Schistosoma* species
*Burkholderia pseudomallei* (melioidosis)	*Orientia tsutsugamushi* (scrub typhus)	Zika	*Strongyloides stercoralis*
Gram-negative bacteria, especially *E. coli* and *Klebsiella*	Hepatitis B	*Trypanosoma cruzi*	*Wuchereria bancrofti* (filariasis)
*Staphylococcus aureus*	Hepatitis C		
*Streptococcal* species	Chikungunya		
Influenza	Varicella zoster		
Dengue	Human immunodeficiency virus		
West Nile virus	Middle East Respiratory Syndrome (MERS)		
	*Leishmania* species		

.

### Bacterial

Tuberculosis (TB) is a leading cause of disease and death worldwide, with an estimated 9 million cases of TB and 1.2 million deaths in 2017.^6^ Diabetes is associated with a threefold increased risk of developing TB,^3^ and increased risk of death or treatment failure in TB.^12^ The GBD group reported diabetes accounting for 10.6% of the TB mortality in HIV-negative people.^13^ In 2017, 790 000 cases of TB were attributable to diabetes,^14^ and the absolute numbers of people with TB-diabetes co-morbidity is now similar to people with TB-HIV co-infection. More than half of the world’s TB cases occur in five countries,^15^ which have significant prevalence rates and total numbers of diabetes cases in adults aged 20–79 years as follows: China (10.9%, 114 million), India (8.8%, 73 million), Indonesia (6.2%, 10 million), Philippines (6.2%, 3.7 million) and Pakistan (6.9%, 7.5 million).^1^ There is evidence that the presence of clinical TB disease drives stress hyperglycaemia, impacting on clinical outcomes and response to treatment.^16^ As rates of diabetes continue to rise, and the TB epidemic continues, there is a pressing need for bidirectional screening in countries facing the double burden of TB and diabetes. Work in India has shown success at screening newly diagnosed TB cases for diabetes,^17^ due to the availability of a simple screening blood test (HbA1c) and the use of existing systems established to screen TB cases for HIV. Screening people with diabetes for TB is more difficult due to the reliance on symptom questionnaires followed by chest x-ray. A blood test to diagnose TB in this setting is highly desirable, but current interferon gamma release assays (IGRAs) do not have sufficient sensitivity and specificity for this purpose. Diabetes is also associated with higher rates of *Mycobacterium leprae*.^18,19^

The greatest increased risk for infection in people with diabetes is seen for the grossly under-recognised tropical disease melioidosis, which is caused by the Gram-negative bacterium *Burkholderia pseudomallei*. People with diabetes have a twelve-fold increased risk of melioidosis, and over half of all cases of melioidosis have diabetes.^20^*B. pseudomallei* is an environmental saprophyte with a predilection for rice paddy fields, and melioidosis is typically seen in adults in middle age and above, often in rice-farming communities. A broad range of clinical presentations are seen, including pneumonia, acute sepsis with bacteraemia, abscess formation in any organ site, chronic subacute disease and latency.^21,22^ Transmission of the bacterium to humans occurs via three routes: inhalational, cutaneous via skin abrasions and ingestion of contaminated drinking water.^23^ The disease is commonly diagnosed in Southeast Asia and Northern Australia, but is now known to be present in 45 countries across tropical regions, with an estimated annual 165 000 cases and 89 000 deaths.^24^ If rates of diabetes continue to rise as predicted, coupled with an increased reliance on older people for rice farming due to exodus of the younger generation to urban areas, then the burden of melioidosis will also rise.

Studies in high-income countries have shown that people with diabetes have high rates of infection from many common bacteria,^25^ but some bacterial species are more frequently reported in association with diabetes. Diabetes is an established risk factor for invasive infection with *Staphylococcus aureus*.^26^*S. aureus* is the commonest cause of tropical pyomyositis, an infection of skeletal muscle featuring intramuscular abscesses that is commonly seen in tropical regions and can account for 1–4% of acute admissions.^27^ Pyomyositis occurs less frequently in temperate zones, where diabetes is a known risk factor.^28,29^ While diabetes has been reported in some case reports of tropical pyomyositis,^30^ further research is needed to establish the association between diabetes and tropical pyomyositis. An association between diabetes and *Salmonella* infections has been reported. A retrospective review of 134 cases of *Salmonella* infection, including 38 cases of *Salmonella* Typhi, revealed that 34% of adults >50 years of age with *Salmonella* infections had diabetes.^31^ Diabetes was associated with around a threefold increased risk of infection with *S. enteritidi*s following exposure in a US hospital outbreak.^32^ People with diabetes have an increased risk of *Klebsiella* infections,^33^ especially *Klebsiella* liver abscess in Asia.^34,35^

Scrub typhus is a febrile illness caused by the Rickettsial group intracellular pathogen *Orientia tsutsugamushi*. Around a million cases a year occur in Asia,^36^ and it is believed to exist in other tropical regions outside Asia.^37^ Diabetes was an independent risk factor for more severe disease in a prospective study of eschar-positive scrub typhus.^38^

It remains to be established why diabetes confers much greater susceptibility to, and worse outcomes for some bacteria than to others. Several of the bacteria most closely associated with diabetes, such as *M. tuberculosis* and *B. pseudomallei*, are predominantly intracellular bacteria. Impairments in phagocyte function and adaptive T cell immunity in diabetes may contribute to increased susceptibility to intracellular pathogens.

Diabetes is associated with antimicrobial resistance (AMR). Diabetes status is associated with increased rates of drug resistance in TB, including multidrug-resistant TB.^39,40^ Besides TB, people with diabetes are over-represented in cohorts with multi-drug-resistant infections,^41^ but there is a lack of evidence at present of higher rates of AMR in bacterial isolates from people with diabetes compared with isolates from non-diabetics. What is clear is that increasing rates of AMR will have a larger impact on people with diabetes, due to their higher risk of infection and increased need for healthcare exposure and interventions.

### Viral

Dengue causes an estimated 101 million clinical cases per year and 37 800 deaths.^6^ An association between diabetes and severe presentations of dengue is now broadly accepted.^42^ However, studies are typically retrospective, often small in nature, and use varying definitions of severe dengue. In a meta-analysis of five case-control studies of acute dengue, diabetes was associated with an increased risk of a severe clinical presentation of dengue compared with either asymptomatic infection or non-severe acute dengue,^43^ although given the limited data, the authors emphasised this was only suggestive of a link. This finding has been supported by further studies in Malaysia^44^ and China.^45^ A systematic review^46^ to evaluate the contribution of non-communicable diseases (NCDs) to the development of severe dengue identified 16 relevant publications and gives a clear overview of this literature, but given the heterogeneity of the studies the authors concluded that the existing literature was inadequate for meaningful estimation of the impact of diabetes and other NCDs on dengue severity. The following year a Canadian group^47^ reported a nearly three-fold higher prevalence of diabetes in 3236 patients in 22 studies with severe dengue than the prevalence of diabetes in 9067 subjects in 13 studies with non-severe dengue. The same analysis found hypertension, heart disease and obesity (conditions interrelated with diabetes) to be significantly more prevalent in severe dengue, and also reported a four-fold increased prevalence of diabetes in severe West Nile fever cases by the same methodology, supporting an earlier study^48^. The authors acknowledge the multiple limitations of this approach, including the heterogeneity of the studies and publication bias for studies included. There is clearly a need for further high-quality epidemiological studies to further define the association. There is some evidence that diabetes is associated with more severe disease in chikungunya disease,^49^ but there is a current lack of data for analysing the relationship with Zika virus.

Other viral infections may be associated with diabetes. People with diabetes are an at-risk group for clinical illness, disease severity and death from influenza,^50^ with a meta-analysis of 234 articles^51^ showing diabetes to be a risk factor for death from pandemic influenza (predominantly H1N1), although there was a lack of high-quality cohort studies to demonstrate this in seasonal influenza. It is estimated that 260 million people are chronic carriers of the hepatitis B virus (HBV),^52^ and HBV has recently been described as a neglected tropical disease.^53^ A number of studies have shown a higher prevalence of HBV in people with diabetes,^54–56^ and people with chronic HBV are reported to have an increased risk of developing diabetes,^57^ although this has not been demonstrated to date in sub-Saharan Africa.^58,59^ In addition, diabetes is associated with disease progression of HBV,^60–62^ which has also been reported for hepatitis C.^63^ Varicella zoster virus (VZV) causes chickenpox as a primary infection which can reactivate as herpes-zoster (shingles), especially in older people. Diabetes is an established risk factor for shingles.^64,65^ Diabetes is considered a risk factor for Middle East Respiratory Syndrome (MERS). A recent systematic review^66^ identified 58 published works for analysis, with several reporting diabetes as a risk factor for infection and death, but meta-analysis to quantitate the increased risk of diabetes across the studies was not possible due to the small number of studies addressing this risk factor.

People living with HIV (PLHIV) who receive combined antiretroviral therapy (ART) now have excellent long-term survival, but increased rates of metabolic disorders such as impaired glucose tolerance, hyperlipidaemia and body morphological changes (lipodystrophy syndrome) have been reported.^67^ Increased rates of insulin resistance in PLHIV could occur due to the pro-inflammatory effects of chronic viral infection, direct effects of ART, and also indirect effects of ART such as dyslipidaemia and body fat distribution changes. Although studies in high-income Western countries have shown inconsistent results as to whether HIV infection increases the risk of T2DM or merely represents earlier diagnosis in a closely monitored population,^68^ there is evidence that HIV increases the risk of diabetes in Asian and African populations. A study in Taiwan suggested PLHIV to be as much as sixfold more likely to develop diabetes than the rest of the population,^69^ and a recent Thai study of PLHIV showed diabetes developed at a younger age compared with the general population.^70^ Increased rates of diabetes have also been reported in PLHIV in South Africa,^71^ Tanzania^72^ and Ethiopia.^71^ Older ART drugs such as stavudine and zidovudine are known to increase the risk of metabolic syndrome and diabetes, and are used less now, so future studies may not confirm this relationship.

### Parasitic

Malaria remains one of the world’s largest causes of mortality from infectious diseases, with an estimated 213 million cases and 720 000 deaths per annum.^6^ In 2010, researchers reported a 46% increased risk of malaria in people with diabetes^73^ in a case-control study in an urban setting in Ghana. The malaria was predominantly *P. falciparum*, asymptomatic and diagnosed by PCR. The comparison of probability of malaria infection was made between patients with diabetes (n=675) and a control group (n=791) comprising patients attending hypertension clinics, patients attending other hospital clinics, and hospital staff. The groups were not matched in that the diabetes group were older by a mean of 7.6 years and had a socio-economic profile associated with greater poverty, and the adjusted odds ratio for diabetes as a risk factor for *P. falciparum* infection was just outside significance in a multivariate analysis adjusting for these parameters (adjusted OR 1.36, 95% CI 0.98–1.90). This report is very interesting, but there have been no further published clinical studies addressing the relationship between diabetes and malaria. If diabetes increases the risk of malaria this would be of huge significance on a global scale. As India is a country with a high burden of both diabetes and *P. vivax* malaria,^74^ one might expect evidence of a relationship between diabetes and vivax malaria to emerge from research in this region. Further prospective studies with well-matched cohorts, and research into the effect on outcomes for people with malaria is needed.

Diabetes has been linked to increased risk of cutaneous^75,76^ and visceral leishmaniasis,^77^ and diabetes and hyperglycaemia were more frequently reported in cardiomyopathy caused by *Trypanosoma cruzi* (Chagas disease) than in controls.^78^ There are a lack of data on the relationship between diabetes and most neglected tropical diseases (reviewed^79^) and prospective studies of people with diabetes in tropical regions are needed to evaluate this.

Some interesting findings have been reported for a lower risk of diabetes following helminth infections including schistosomiasis, strongyloides and filariasis, as reviewed by Berbudi and colleagues.^80^ The mechanism for such a protective effect could be by helminth infection inducing a shift towards type 2 (anti-inflammatory) immune responses, and reducing chronic low-grade inflammation. A mouse model^81^ demonstrated greater insulin sensitivity following *S. mansoni* infection in mice fed a high-fat diet, with an increase in type 2 cytokines and the ratio of M2 to M1 macrophages in white adipose tissue. Further research is evaluating whether helminth-derived molecules could be developed as novel therapeutic approaches to diabetes and metabolic syndrome.^82^ In addition, a prospective randomised-control trial of helminth eradiation by albendazole therapy in Indonesia^83^ is underway evaluating the impact of helminth infections on insulin resistance.

## Mechanisms of increased susceptibility to infection in diabetes

The mechanisms by which diabetes confers altered susceptibility to infections are likely to be via multiple effects on the human immune system.

### General and systemic effects of diabetes on the immune system

People with diabetes have altered skin flora, including increased colonisation with *S. aureus*.^84^ Breaches in the skin’s integrity as a physical barrier to infection occur more commonly in diabetes due to the impact of chronic hyperglycaemia on peripheral nerves and vascular supply. Diabetes and its treatment has an impact on the composition of the gut microbiome,^85^ and the pivotal role of the gut microbiome in modulating human innate and adaptive responses via pathogen recognition receptor pathways and secretion of immunomodulatory molecules by gut bacteria is now emerging.^86^ In vitro, mild hyperglycaemia may favour pathogen growth, and while the contribution of this to human susceptibility to infection is unknown, glycosuria favours urinary tract infections. In addition, hyperglycaemia has a number of immunosuppressive effects, including impairment of neutrophil degranulation, complement activation and phagocytosis.^50^ Hyperglycaemia does not, however, appear to be a key mechanism for the increased susceptibility to TB.^87^ Diabetes is associated with endothelial dysfunction, oxidative stress and chronic inflammation.^88^ In infections like dengue, this may support a shift towards an excessive pro-inflammatory response, leading to the cytokine storm, shock, vasculopathy and coagulopathy that feature in severe dengue.^47^ Finally, people with diabetes have higher rates of hospital admissions,^1^ which exposes them to hospital-acquired infections and the risk of AMR.

There is overlap between the spectrum of pathogens that people with diabetes have, notably increased susceptibility to such as *S. aureus* and invasive fungi, and the infections seen in chronic granulomatous disease (CGD),^89^ which is congenital deficiency in phagocytic function. This suggests impairment of neutrophil and macrophage function in people with diabetes as a key mechanism of susceptibility. This is supported by studies showing impaired neutrophil migration, phagocytosis and intracellular killing in the host response to *B. pseudomallei* in people with diabetes.^90^ However, people with CGD are not known to have increased susceptibility to viral infections, and therefore this impairment of phagocyte function is unlikely to be the only cause of increased susceptibility to infection in diabetes.

### Antibody responses

The literature on humeral responses to infection in people with diabetes does not suggest that poor antibody response is a dominant mechanism for the increased susceptibility to infection seen in diabetes. Most studies support adequate induction of antibodies in response to licenced antibody-inducing vaccines in people with diabetes^91–93^. Higher antibody responses to influenza vaccine were seen in a US study of elderly people with diabetes compared with elderly non-diabetic people,^94^ and antibodies induced by natural exposure to melioidosis were higher in those with diabetes.^95^ Such higher responses in diabetes could be due to chronic hyperactivation of the innate immune response in T2DM resulting in polyclonal B-cell stimulation and enhanced antibody production to stimuli.^96^

### Cellular responses

Beyond neutrophil changes, people with diabetes have alterations in the function of several cell types including macrophages, Natural Killer cells, CD4 T cells and CD8 T cells function.^97^ T cell responses are known to be important in host defence against intracellular pathogens, with intracellular antigens presented to CD8 T cells via the MHC Class 1 antigen presentation pathway, and digested antigens from both extracellular and intracellular sources presented to CD4 T cells via the MHC Class 2 system. HIV results in reduction of CD4 T cells, and the huge increased risk of TB seen in advanced HIV demonstrates the importance of CD4 cells in defence against TB. There is evidence of impaired antigen-specific T cell responses in people with diabetes in response to early TB^97^, B. pseudomallei^20^ and VZV^98^, although in established TB the cellular response appears to be higher in diabetes.^97^ In addition, differences in the regulation and orchestration of the immune response are seen in dengue.^99^

For further detailed discussion of immune mechanisms, the reader is referred to a number of excellent reviews of immune impairment in diabetes.^97,100^ Overall, diabetes can impair the immune system at a systemic, cellular and molecular level, but it is the alteration of T cell function that is most amenable to boosting with targeted vaccination strategies.

Acute infection is known to lead to hyperglycaemia as a consequence of the stress-response activation of the hypothalamic-pituitary-adrenal axis to increase secretion of cortisol and other hormones which promote peripheral insulin resistance, alongside alteration of insulin-receptor signalling by pro-inflammatory cytokines (reviewed^101^). Sepsis-related hyperglycaemia may be a risk factor for future development of T2DM,^101^ and stress hyperglycaemia induced by chronic infections such as TB may contribute to the global burden of diabetes.^16^

## Interventions

Concerted international efforts to stem the tide of advancing diabetes in LMIC are urgently required. Implementation of evidence-based approaches that are effective at both prevention and early intervention for diabetes are needed. Such approaches require behavioural changes in diet and physical inactivity, raising public awareness of diabetes, and convincing policy-makers and the public alike of the benefits of early diagnosis for disease reversal and management. One of the targets for the UN’s Sustainable Development Goal 3 (Good Health and Well-being) is to reduce by one-third premature mortality from NCDs through prevention and treatment by 2030, with action on diabetes essential. Actions include implementation of National Diabetes programmes and extension of health promotion activities.^1^

Tackling the interaction between diabetes and infection requires a greater understanding of the immune mechanisms underlying the altered susceptibility to infection seen in diabetes, and knowledge of how therapeutic management of diabetes impacts on risk of infection. It is likely that tight control of hyperglycaemia lowers the risk of infection, although this has yet to be comprehensively demonstrated in prospective studies. Current international treatment guidelines for T2DM are based on research conducted in high-income countries focussed on preventing adverse cardiovascular outcomes and early death. There is a lack of evidence on which to base guidelines for people living in LMIC, and choice of glucose-lowering therapy may impact on infection risk and outcomes. For example, there is emerging evidence for beneficial infection outcomes in people with diabetes taking metformin compared with other therapies,^102–104^ and glyburide/glibenclamide has been associated with anti-inflammatory properties and lower mortality in melioidosis.^105^

Vaccination remains the cornerstone in controlling infectious diseases, and people with diabetes are prioritised as a high-risk group for vaccination against a range of pathogens including pneumococcus, influenza and VZV. Development of highly efficacious vaccines against intracellular infections such as TB, melioidosis and leishmaniasis, to which people with diabetes are at greater risk, is an important priority, and progress requires consideration of how to overcome the specific immune impairments seen in diabetes. Characterising the impact of diabetes on protective immunity is particularly important for melioidosis, where more than half of all cases occur in people with diabetes, and cost-effective implementation of a successful vaccine is likely to involve targeting this group.^106^*B. pseudomallei* therefore represents an exemplar pathogen for defining the immune deficits in diabetes, and how to overcome them.

## Conclusions

The collision of diabetes and the world’s global infections is a highly neglected area. More research to clearly define the epidemiology and illuminate successful interventions needs prioritising. The majority of the evidence for associations between diabetes and specific infections relies on the use of relatively small retrospective case-control studies, and meta-analysis approaches are limited by the heterogeneity of patient-level factors across studies. Diabetes is interrelated with obesity, hypertension and cardiovascular diseases that share similar risk factors and are each linked to adverse disease outcomes, rendering elucidation of the precise contribution of diabetes to excess morbidity and mortality difficult. The causal interaction between diabetes and infection rates and outcomes is also challenging. For some chronic viruses such as HBV and HIV, the association with diabetes may represent increased risk of diabetes by pathogenesis mechanisms resulting in insulin resistance related to chronic inflammation, rather than the other way around.

High-quality, large prospective epidemiology studies are needed to quantitate the problem, alongside randomised controlled trials of interventions to define optimal treatment strategies in diabetes. Raising awareness of the interaction of diabetes and infection with policy-makers, and ensuring the engagement of social scientists, health economists and the pharmaceutical industry in developing new strategies to fight this double burden in LMIC is vital.
